# Structural Mapping of Surveillance Data Reveals Conservation of NNI Binding Site in RSV L Protein

**DOI:** 10.3390/pathogens15010085

**Published:** 2026-01-13

**Authors:** Ruchin Patel, Edward Murray, Debbie D. Nahas, Mahdieh Yazdani, Brett Ambler, Nicholas Murgolo, John A. Howe

**Affiliations:** Merck & Co., Inc., Rahway, NJ 07065, USA

**Keywords:** RSV, polymerase, PRNTase, MRK-1, NNI, resistance, surveillance

## Abstract

Respiratory syncytial virus (RSV) remains a leading cause of lower respiratory tract infections (LRTIs) and infant mortality worldwide. Despite recent advances in prophylactic interventions, effective therapeutics for active RSV infection are still lacking. Small molecule non-nucleoside inhibitors (NNIs) targeting the RSV L protein, particularly its polyribonucleotidyltransferase (PRNTase) domain, represent a promising antiviral strategy. Here, we evaluate the genetic variability of the PRNTase domain and the binding pocket of two NNIs, MRK-1 and MRK-2, to assess the potential for preexisting resistance. A comprehensive analysis of 28,140 RSV L protein sequences from NCBI Virus and GISAID EpiRSV databases revealed low overall variability within the PRNTase domain and near-complete conservation of the MRK-1/2 binding pocket. Resistance-associated mutations identified through in vitro dose-escalation studies localized to this pocket but were absent in global sequence datasets. These findings support the PRNTase domain as a genetically stable and viable target for NNI-based RSV therapeutics and suggest a low likelihood of preexisting resistance among circulating strains.

## 1. Introduction

Respiratory syncytial virus (RSV) is a leading cause of acute lower respiratory tract infection in infants, young children, elderly adults, and adults with comorbidities worldwide, producing a clinical spectrum that ranges from mild upper respiratory disease to life-threatening bronchiolitis and pneumonia, with the highest risk of severe outcomes and death in infants under six months of age [1,2,3]. Before widespread availability of RSV prophylaxis and vaccines, the global pediatric burden was substantial: in 2019 an estimated 33.0 million RSV-associated acute lower respiratory tract infections (LRTIs), 3.6 million RSV-associated LRTI hospital admissions, and more than 100,000 deaths in children under five were attributed to RSV [3]. Post-COVID changes in viral circulation have not eliminated this burden: 2022 saw persistently elevated RSV mortality in young children at 9.76 deaths per 100,000 children under 5, and the Global Burden of Disease Study continues to register RSV among leading causes of pediatric morbidity and mortality, with the burden disproportionately concentrated in low- and middle-income countries (LMICs) [3,4]. These data underscore an ongoing, unmet medical need for effective RSV therapeutics to supplement the current vaccines and prophylactic antibodies, particularly options that can treat established infection and reduce severe outcomes [5,6,7,8,9,10,11,12].

The RSV viral polymerase complex is a compelling antiviral target because it is essential for both viral transcription and genome replication and contains multiple enzymatic functions that can be inhibited pharmacologically. Central to this complex is the large polymerase (L) protein. The multifunctional L protein is composed of several discrete domains: an RNA-dependent RNA polymerase (RdRP) domain that mediates RNA chain synthesis; a polyribonucleotidyltransferase (PRNTase) domain which facilitates 5′ cap addition to viral mRNAs; a connector domain (CD); a methyltransferase (MT) domain involved in cap methylation; and a C terminal domain (CTD) [13,14,15,16,17,18,19]. High-resolution structural studies have defined these domain arrangements and suggested conformational changes that underlie transitions between initiation, elongation, and mRNA capping during the viral transcription–replication cycle [16,17,18].

Multiple antiviral strategies targeting the L protein have been explored [8,20,21,22]. Nucleoside analogues inhibit the RdRP domain by acting as chain terminators or mutagens [23,24]. In contrast, non-nucleoside inhibitors (NNIs) act allosterically by binding to regulatory pockets within the L protein, typically the CD and PRNTase domain, to block conformational transitions required for distinct stages of polymerase function [25,26,27,28,29,30,31]. These different mechanisms offer complementary opportunities to suppress viral replication. However, a critical consideration in antiviral development is the potential for preexisting resistance due to natural polymorphisms in circulating viral populations. Sequence variation at or near drug-binding sites can compromise antiviral efficacy, as demonstrated by the clinical failure of suptavumab, a monoclonal antibody targeting the RSV F protein, due to widespread circulation of RSV B strains harboring mutations that abrogated antibody binding [8,29].

Given the importance of assessing preexisting resistance, we focused on a class of NNIs exemplified by MRK-1 and MRK-2, which target a conserved pocket within the PRNTase domain of the RSV L protein [28,29]. Prior PKPD evaluation of MRK-1 in cotton rat and African green monkey models of RSV infection showed lack of drug-specific resistance mutation at I1381 and L1421 [30]. Our study evaluated sequence variability of the PRNTase domain and the MRK-1/2 binding pocket across globally circulating RSV strains. We performed a comprehensive residue-level analysis of 28,140 RSV L protein sequences obtained from public databases, mapped observed variants onto structural models of the polymerase, and conducted in vitro dose escalation experiments to identify resistance-conferring mutations. Our findings demonstrate that the PRNTase domain, and specifically the MRK-1/2 binding pocket, is highly conserved, with minimal natural variation and no evidence of preexisting resistance mutations in circulating strains. These results support the continued development of PRNTase-targeting NNIs as promising therapeutic candidates for RSV infection.

## 2. Materials and Methods

### 2.1. Sequence Collection

We retrieved 13,499 RSV L protein sequences from the NCBI Virus browser by filtering the dataset for RSV proteins with sequence lengths of 2150 to 2170 [32]. The subtypes for the NCBI sequences were identified using the metadata, including organism and isolate names. We also retrieved 45,550 RSV A and 33,729 RSV B genome sequences from GISAID EpiRSV [33]. To isolate and identify whole L protein sequences, the genomes were compared with L protein sequences from reference sequences (RSV A: NC_001803; RSV B: NC_001781) with the NCBI BLAST+ (2.2.28) command line application, and hits that covered the whole sequence were extracted to be analyzed [34].

### 2.2. Sequence Year and Location Isolation

The location and collection-year data were pulled from the metadata associated with the sequences within the NCBI and GISAID databases. For sequences without explicit location and collection-year metadata entries, the information was pulled from other metadata or associated publications where available. All location data that was at the city level was mapped back to the country of origin.

### 2.3. Residue Variation

For each of the four groups of proteins, the sequences were aligned using the ClustalO command line tool [35]. The residue frequency at each position was calculated using the EMBOSS Prophecy command line application [36]. The frequency tables were used to identify consensus residues (residues that show up in ≥50% of sequences with non-ambiguous amino acids at that position), major variants (<50% and ≥1%), and minor variants (<1% and ≥0.1%).

### 2.4. Mapping the Residue Variation with Respect to the Binding Site

The cryo-EM structure of the truncated RSV L protein bound to the inhibitor MRK-1 has been solved (PDB ID: 8FPI resolution 2.52 A with domain boundaries noted in reference) [31]. The 5 A binding site was defined using PyMOL (3.1.6.1) with the command “elect nearligm byres chain A within 5 of 9001”. The 31 residues in the binding site are P1002, H1120, G1218, V1219, T1220, S1221, I1241, S1266, L1337, H1338, R1345, P1346, F1349, T1365, I1368, N1369, L1372, T1373, Y1376, G1377, D1378, E1379, D1380, I1381, D1382, I1383, V1384, F1385, Q1386, C1388, and M1422. To obtain the docking pose for the MRK-2 structure, we used a closely related cryo-EM bound structure in which the bound small molecule had a few atom differences with respect to MRK-2, albeit with the same binding site as MRK-1. This provided strong confidence in the predicted pose in this pocket. Subsequently, these structures were used to map the residues with major and minor variations while highlighting their positions with respect to the binding site. MOE and PyMol were used for docking and visualization analysis [37,38].

### 2.5. Potency Evaluation

For all compound potency assays, we used an RSV GFP reporter virus that was described previously [29]. MRK-1 was assayed against RSV A2 GFP and MRK-2 against RSV B Wash GFP. The compounds were dissolved in DMSO and titrated in an 11-point 3-fold serial dilution in a 96-well tissue culture plate. The dilutions were mixed with 2 × 10^4^ Hep-2 cells in DMEM with 10% heat inactivated fetal bovine serum (FBS) infected at a 0.1 MOI. After 2 days of incubation at 37 °C, the fluorescence was measured using an Acumen Cellista plate reader. EC_50_ and EC_90_ concentrations were calculated by fitting a dose-response curve to the data using GraphPad Prism (10.2.2).

### 2.6. Resistance Selection

**Scheme 1:**
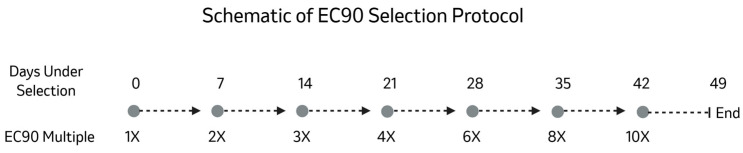
EC90 resistance selection gradient. RSV-infected cells were initially exposed to the inhibitor at 1X EC90, with the concentration increased in stepwise multiples of EC90 every 7 days. Virus was passaged twice per week at the prevailing concentration. An RSV–GFP reporter virus enabled visualization of infected wells and identification of viral populations that persisted under selection to 10X EC90 for subsequent genotyping.

(Detailed in Scheme 1). 2 × 10^4^ HEp-2 cells in 10% FBS-DMEM were added to each well of a 96-well cell culture plate. DMSO solutions of MRK-1 and MRK-2 were added to each well of the first 11 columns at their respective EC_90_ concentrations. The plates were infected with RSV A2 GFP and RSV B Wash GFP, respectively, at an MOI of 0.1. After 3 to 4 days of incubation at 37 °C, the fluorescence was read in an Acumen Cellista plate reader. The plate was then passaged by preparing plates with 2 × 10^4^ HEp-2 cells/well and MRK-1 or MRK-2 and transferring 10 μL of supernatant from each well on the old plates into the corresponding well on the new plates. The concentration was increased on every other passage going from 1× EC_90_ to 2×, 3×, 4×, 6×, 8× and finally 10× the EC_90_.

### 2.7. Sequencing

The viral RNA for each resistant well was extracted using the QIAGEN QIAamp Viral RNA Mini Kit. The RNA was converted to cDNA using the SuperScript IV UniPrime 1-Step RT-PCR kit. The PCR and sequencing primers used were TGACACAATTCAAAATTTTCTACAACA (F) and ATTCCTCCAAGATTAAAATGATAACTTTAG (R) for RSV A2; TCCACAAAAGCATAACCATAAGCA (F) and TGTCTCGTTGTGTTGTAAATGCA (R) for RSV B Wash.

### 2.8. Variant Potency Shift

RSV variants were generated by mutating the pSmart RSV A2 GFP via Gibson assembly. The introduction of the mutation was verified by sequencing. The recombinant viruses were recovered through transfection into BSR-T7 via Mirus TransIt-LT1 of a mixture of the mutated pSmart BAC along with pcDNA3.1, M2-1, N, P, and L in a 2:2:2:2:1 ratio.

DMSO solutions of MRK-1 and MRK-2 were titrated in an 11-point 3-fold serial dilution in a 96-well tissue culture plate. The dilutions were mixed with 2 × 10^4^ Hep-2 cells in DMEM with 10% heat inactivated fetal bovine serum (FBS) infected with 10 μL of supernatant from the resistant wells. After 2 days of incubation at 37 °C, the fluorescence was read in an Acumen Cellista plate reader. EC_50_ and EC_90_ concentrations were calculated by fitting a dose-response curve to the data using GraphPad Prism.

## 3. Results

### 3.1. Sequence Gathering

We pulled 13,499 L protein sequences from NCBI and 79,279 genomes (45,550 RSV A and 33,729 RSV B) from GISAID (EPI_SET ID: EPI_SET_251031go). NCBI did have L protein sequences available, but they were not all assigned to a subtype. After checking the other metadata, there were 6284 RSV A and 5582 RSV B sequences; the rest had insufficient data to conclude their subtype and were therefore discarded.

GISAID has a subtype assigned to all RSV genomes but does not have the L protein sequences readily available. After running a BLAST to extract the L protein sequence from their genome sequences, 13,279 RSV A and 14,945 RSV B sequences were identified as complete. Due to the lack of metadata to cross-reference between the two databases, it was not possible to determine the overlap between GISAID and NCBI submissions. For this reason, we have kept the sequences from each database in distinct groups, creating four (4) datasets (Table S1).

**Figure 1:**
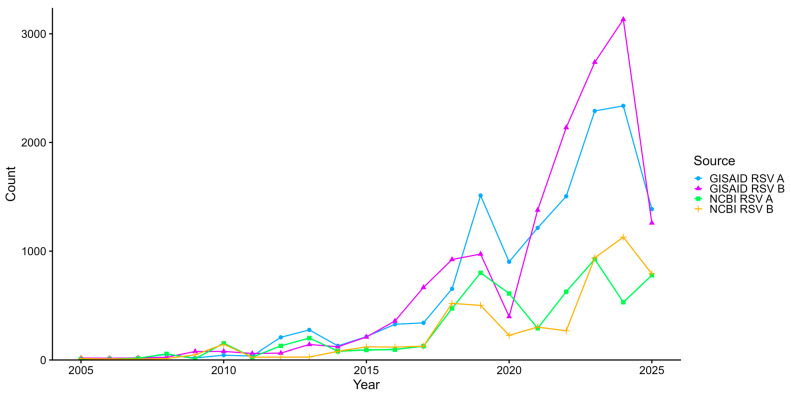
RSV L Protein Sequences by Collection Year. The collection year of L protein sequences used from 2005 onwards. The count for sequences pre-2005 is 214 RSV A, 190 RSV B from GISAID and 190 RSV A, 69 RSV B from NCBI.

**Figure 2:**
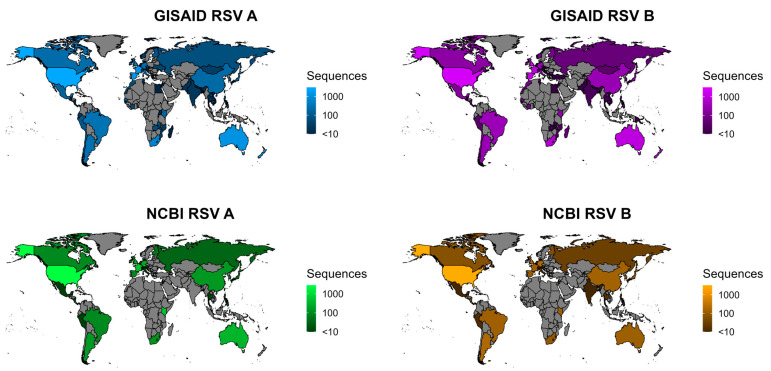
RSV L Protein Sequences by Country. Number of L protein sequences obtained for each subtype by database from each country.

These datasets are contemporary, with 56.4% of the sequences collected between 2022 and 2025 (Figure 1). The data are unfortunately not globally diverse, with LMICs being underrepresented (Figure 2). The Americas, Western Europe, and East Asia have good sequencing coverage. Australia, New Zealand, South Africa, and Kenya individually have high sequencing coverage, but the rest of Africa and Oceania have little to no sequencing.

### 3.2. Low Variance in PRNTase

To understand which regions of the RSV L protein are conserved across the global population, the datasets were aligned, and variability was calculated at the positional level (Tables S2–S5). For each position, the amino acids were categorized into 3 categories: consensus, accounting for 50+% of the residues aligned to that position; major variant, making up at least 1% but less than 50% of the aligned residues; and minor variant, making up between 0.1% and 1% of the residues. All amino acids that made up less than 0.1% of the residues aligned to that position were considered insignificant. If the consensus amino acid at a position makes up less than 90% of the residues aligned to that position, that position is labeled as highly variable. Most positions have low variability, with only 11 (0.51%) positions in RSV A and 10 (0.46%) positions in RSV B being labeled as highly variable (Table 1). For both RSV A and RSV B, there are 4 highly variable positions in the RdRP, 4 in the CD and 1 in the CTD. There are 2 highly variable positions in the MT in RSV A and 1 in RSV B. The PRNTase is the least variable domain of the L protein, with no highly variable positions in either subtype.

The PRNTase domain is also the least covered by significant variants (Table 2). Minor variants cover 0.14–0.22% of the whole RSV L protein compared to 0.09–0.14% coverage of the PRNTase domain. Excluding the NCBI RSV B data, major variants cover 0.02–0.04% of the RSV L protein. For the NCBI RSV B dataset, that number jumps to 0.14%, but the variant coverage for the PRNTase domain remains at 0.01% for all groups. The PRNTase domain shows low variation as compared to the rest of the L protein.

### 3.3. Low Variance in MRK-1/MRK-2 Binding Pocket

**Figure 3:**
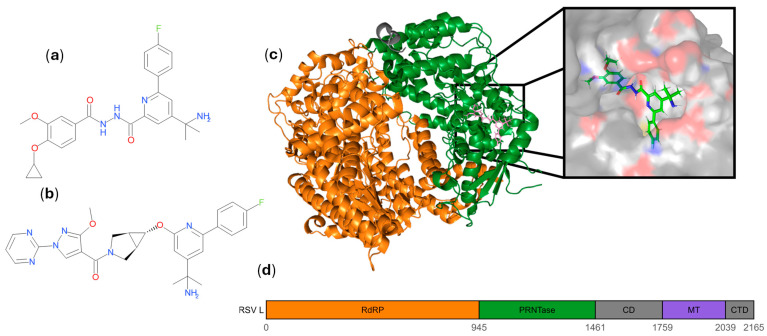
NNI binding site in the RSV L protein. (**a**) Structure of MRK-1. (**b**) Structure of MRK-2. (**c**) Cryo-EM structure of the truncated RSV L protein bound to MRK-1, colored by domain and zoomed-in on the MRK-1/2 binding pocket, colored red for oxygen and blue for nitrogen. (**d**) Diagram of RSV L protein domains with the functional domains colored (boundaries from reference [31]).

**Figure 4:**
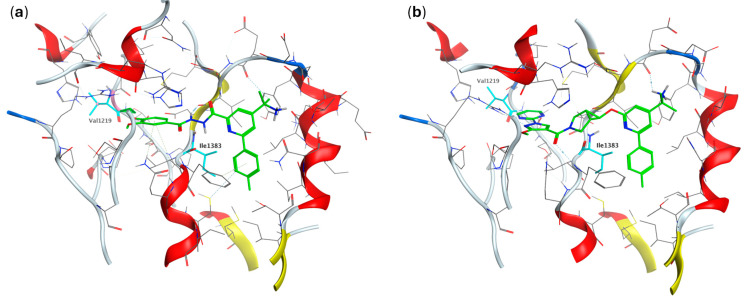
Minor Variants in RSV A MRK-1/2 Binding Pocket. (**a**) Cryo-EM model of MRK-1 and (**b**) docked model of MRK-2 bound to the truncated RSV L protein, highlighting residues with minor variants in the RSV A surveillance data in cyan.

MRK-1 binds in the PRNTase domain (Figure 3) of the RSV L protein. MRK-2 is structurally close to MRK-1 and binds to the same binding site as MRK-1. The docked model shows MRK-2 fits well into the pocket and interacts with similar residues. Though there are minor variations in the residue positions, the overall shape and structure of the pocket is well maintained (Figure 4).

**Figure 5:**
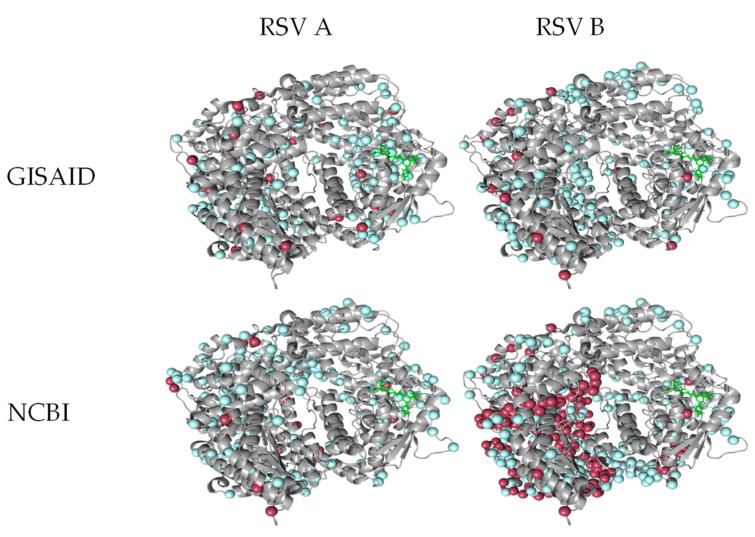
Variation Within the RSV L Protein. Structure of the truncated RSV L protein with spheres to indicate positions with major (dark red spheres) and minor (light cyan spheres) variants for RSV A (**left**) and RSV B (**right**) according to data from GISAID (**top**) and NCBI (**bottom**).

None of the residues directly interacting with the small molecule in the binding site show significant variation in the surveillance data (Figure 5). Inspecting the binding pocket residues also reveals that the consensus amino acid is identical between RSV A and RSV B with no major variants (Table S6). There are, however, only two (2) minor variants within the binding pocket in the surveillance data (V1219I [0.2%] and I1383V [0.1%]) for RSV A (Figure 4). While the I1383 side chain has a hydrophobic interaction with the ligand in the binding site, both observed variants are considered conserved and are expected to have minimal effect on the binding affinity of the small molecule inhibitor. Therefore, it can be inferred that the MRK-1/2 binding pocket is highly conserved, and the lack of variation in the binding pocket implies that the collected strains should not show resistance to either NNI, assuming all resistance-conferring mutations occur in the binding pocket.

### 3.4. Dose Escalation Selects for Resistance Mutations Within the Binding Pocket

**Figure 6:**
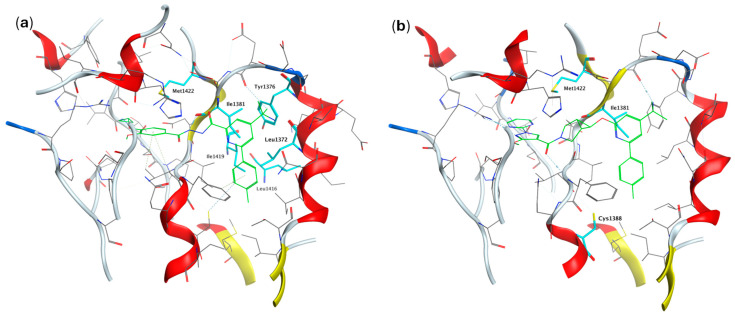
Potentially Resistance-Conferring Mutations Identified Through Dose Escalation. Diagram highlighting in cyan and labeling the residues where mutations occurred during dose escalation experiments that likely confer resistance against (**a**) MRK-1 and (**b**) MRK-2.

To find mutations likely to confer resistance, we performed a dose escalation experiment. Of the eighty-eight (88) replicates, MRK-2 selected for resistance in eighteen (18) and MRK-1 for thirty-three (33). Six (6) of the MRK-1 resistant samples showed double mutations. Sequencing these resistant samples showed there were mutations at three (3) distinct positions across the samples selected against MRK-2 and at ten (10) for those against MRK-1 (4 only in samples with multiple mutations). There were two (2) positions where resistant mutants were selected against both NNIs (I1381 and M1422). MRK-2 also selected for a mutant in C1388, and MRK-1 selected for potentially resistance-conferring mutations at L1372, Y1376, L1416, and I1419 (Figure 6). Of these seven (7) positions, five (5) are in the binding pocket, directly interacting with the small molecule inhibitor (the first shell), and the other two (2) are interacting with the residues making up the binding site (the second shell). Neither of the second shell positions show significant variation in the surveillance data (Tables S2–S5).

**Figure 7:**
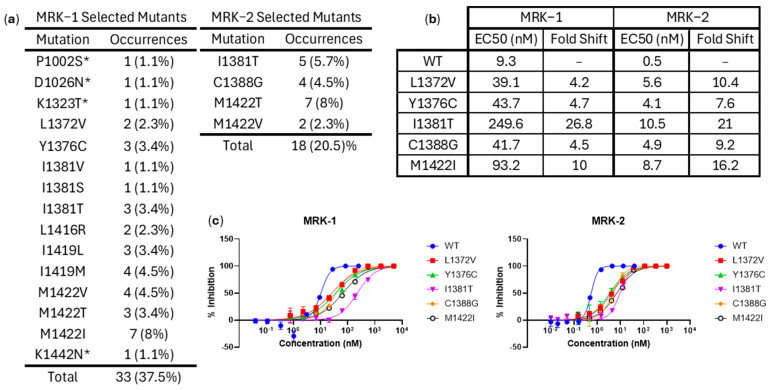
Resistant Mutants Identified Through Dose Escalation. (**a**) List of mutants isolated from dose escalations against MRK-1 and MRK-2 with those that only showed up as part of a double mutation virus marked *. (**b**) EC_50_ of MRK-1 and MRK-2 against specific mutants compared to those against RSV A2. (**c**) Potency curves for MRK-1 and MRK-2 against specific mutants and RSV A2.

Five of the generated mutations were selected for evaluation to verify their resistance to the NNIs. I1381T and M1422I were particularly selected because they arose for both NNIs in the dose escalations. C1388G only showed up in the escalation against MRK-2, while L1372V and Y1376C only showed up in the escalation against MRK-1. These mutations were expressed in recombinant viruses. All 5 of these mutants showed resistance to the NNIs, with the EC_50_ of each being ≥3 fold greater than that of the wild-type RSV A2. The fold shift against MRK-2 was generally larger than against MRK-1, but MRK-2 was more potent against every tested virus (Figure 7).

Most of the positions identified through the dose escalations are within the identified binding pocket. None of the identified positions show significant variants.

## 4. Discussion

The currently available RSV surveillance data show that the PRNTase domain of RSV L protein, and the MRK-1/2 binding pocket in particular, is highly conserved. There are thousands of L protein sequences from both RSV A and B, with the majority being contemporary. GISAID has more than double the available RSV L protein sequences compared to NCBI, and much of that discrepancy is explained by GISAID having more sequence submissions post-2020 than NCBI. There are more RSV nucleotide sequences available than RSV L protein sequences, as submitted sequences are often partial genomes and the L protein is not typically a priority target for sequencing in genotyping and surveillance studies of RSV. With the available full-length L protein sequences, we have demonstrated that the residues within 5 Å of the binding pocket exhibit minimal variation across the current global RSV population.

To confirm that low variation in the MRK-1/2 binding pocket means that there is no preexisting resistance to these NNIs in the circulating RSV strains, we performed dose escalation experiments against each NNI to select for resistance-conferring mutations. Some mutations were selected during both dose escalations, and the rest were selected during only individual assays. A handful of these mutations were tested and shown to confer resistance to both NNIs. Most of these mutations occurred within the MRK-1/2 binding pocket. The mutations that occurred outside but close to the pocket are at positions that show no variation in the circulating RSV. None of the selected mutants were seen as variants in the surveillance data. Given this data, it is highly unlikely that there is any preexisting resistance to these PRNTase-targeting NNIs within the global RSV population.

There are limitations to this analysis. Though the sequences are contemporary, they cannot be said to be globally diverse. The sequences mostly originate from high-income countries, with data from LMICs being comparatively sparse. This is important, as the burden of RSV is higher in LMICs. This perennial problem extends past this study. LMICs are understudied and have poor surveillance. Without investment in public health surveillance in these countries, it will be challenging to gain a comprehensive understanding of the complete variation landscape of any virus. However, since there are data from Kenya, Egypt, South Africa, India, and Vietnam, countries that are and border LMICs, some of the variation prevalent in LMICs is represented in the dataset.

Another limitation, and potential avenue for future research, is that the list of resistance-conferring mutations generated from the dose escalations is not exhaustive. Only one dose escalation was performed against each NNI, while each assay has eighty-eight (88) replicates, which is not enough to find every resistance-conferring mutation. This is demonstrated by the presence of unselected mutations in the dose escalation against MRK-1, which exhibited resistance, and similarly for MRK-2. Although more resistance-conferring mutations are likely to be identified with further dose escalation assays, they are likely to be located within the binding pocket or adjacent to it. Neither limitation impacts the conclusion of this investigation.

This form of surveillance analysis enables the further development of NNIs targeting PRNTase without concern that existing strains may be resistant. Such foresight is enabled by the increases in public health surveillance and data sharing through publicly accessible databases that have occurred after the COVID-19 pandemic.

## 5. Conclusions

A substantial amount of data indicates high conservation within the RSV L protein PRNTase domain, particularly within the MRK-1/2 binding pocket. No mutations that could confer resistance to either MRK-1 or MRK-2 have been identified in the surveillance data. As such, this structurally defined binding pocket is a promising target that is unlikely to have any RSV strains with pre-existing resistance.

## Figures and Tables

**Table 1:** Highly Variable Positions. Positions where the most common residue shows up in <90% of all sequences. The percent prevalence of each residue at each position is displayed within parentheses. N/A indicates that the position has a consensus residue with a >90% prevalence in the sequences from that database.

Subtype	Position	GISAID			NCBI		
		Consensus	Major Variant	Minor Variant	Consensus	Major Variant	Minor Variant
A	143	N (66.28)	D (33.61)	–	N (67.83)	D (32.04)	–
	179	T (62.43)	A (3.61) S (33.4)	I (0.24) K (0.13) L (0.14)	T (64.15)	A (3.51) S (31.67)	I (0.34)
	598	H (89.16)	Y (10.84)	–	H (81.89)	Y (18.09)	–
	835	L (75.87)	M (24.08)	–	L (79.01)	M (20.91)	–
	1653	I (63.92)	V (35.97)	–	I (65.57)	V (34.31)	M (0.1)
	1661	K (66.38)	N (33.42)	S (0.17)	K (66.61)	N (33.08)	S (0.29)
	1723	S (76.87)	C (2.19) G (19.55) N (1.31)	–	S (78.79)	C (4.69) G (14.81) N (1.64)	–
	1725	G (73.37)	D (4.09) E (22.28)	R (0.15)	G (66.44)	D (10.35) E (22.77)	R (0.26)
	1789	N/A			S (89.32)	F (10.53)	T (0.11)
	1943	V (80.18)	D (19.14)	A (0.19) I (0.4)	V (83.99)	D (15.16)	A (0.38) I (0.35)
	2163	Y (78.02)	H (3.21) N (18.7)	–	Y (70.51)	H (6.93) N (22.35)	–
B	146	N/A			G (88.48)	D (11.08)	S (0.33)
	195	N/A			C (85.16)	M (3.92) R (9.27) Y (1.55)	–
	570	R (56.81)	K (43.19)	–	R (55.96)	K (44.04)	–
	715	N/A			V (89.34)	I (10.66)	–
	1479	A (84.73)	V (15.25)	–	A (79.57)	L (3.09) V (17.32)	–
	1712	N/A			A (88.98)	T (10.96)	–
	1716	V (86.71)	I (12.29)	A (0.17) M (0.73)	V (82.44)	I (13.98) M (1.02) N (2.36)	A (0.15)
	1759	K (57.2)	R (42.78)	–	K (52.43)	R (47.57)	–
	1965	V (85.72)	I (13.42)	A (0.81)	V (73.69)	I (26.06)	A (0.22)
	2162	N/A			L (89.48)	F (10.22)	I (0.2)

– indicates no minor variant at that position.

**Table 2:** Positions With Variants. The number of positions where there is at least 1 major variant (a residue with a prevalence between 1% and 50%) or at least 1 minor variant (a residue with a prevalence between 0.1% and 1%).

Domain	Positions with ≥1 Major Variant				Positions with ≥1 Minor Variant			
	RSV A		RSV B		RSV A		RSV B	
	GISAID	NCBI	GISAID	NCBI	GISAID	NCBI	GISAID	NCBI
RdRP	32 (0.03%)	36 (0.04%)	22 (0.02%)	175 (0.19%)	114 (0.12%)	129 (0.14%)	143 (0.15%)	258 (0.27%)
PRNTase	5 (0.01%)	2 (0%)	3 (0.01%)	5 (0.01%)	48 (0.09%)	73 (0.14%)	50 (0.1%)	44 (0.09%)
CD	8 (0.03%)	28 (0.1%)	12 (0.04%)	114 (0.39%)	33 (0.11%)	80 (0.27%)	74 (0.25%)	73 (0.25%)
MT	25 (0.09%)	17 (0.06%)	11 (0.04%)	11 (0.04%)	68 (0.24%)	55 (0.19%)	82 (0.29%)	74 (0.26%)
CTD	10 (0.08%)	6 (0.05%)	2 (0.02%)	3 (0.02%)	41 (0.32%)	21 (0.17%)	32 (0.25%)	26 (0.2%)
All	80 (0.04%)	89 (0.04%)	50 (0.02%)	308 (0.14%)	304 (0.14%)	358 (0.17%)	381 (0.18%)	475 (0.22%)

## Data Availability

The surveillance sequences are available at GISAID under the EPI Set ID EPI_SET_251031go.

## Supplementary Materials

The following supporting information can be downloaded at: https://www.mdpi.com/article/10.3390/pathogens15010085/s1, Table S1: All accession numbers for sequences used; Table S2: Variation in RSV A L protein sequences collected from GISAID EpiRSV at each position; Table S3: Variation in RSV A L protein sequences collected from NCBI Virus at each position; Table S4: Variation in RSV B L protein sequences collected from GISAID EpiRSV at each position; Table S5: Variation in RSV B L protein sequences collected from NCBI Virus at each position; Table S6: Variation in MRK-1/2 Binding Pocket.

## Abbreviations

The following abbreviations are used in this manuscript:

RSV	Respiratory syncytial virus
LRTI	Lower respiratory tract infections
LMIC	Low- and middle-income country
L	Large polymerase protein
RdRP	RNA dependent RNA polymerase
PRNTase	Polyribonucleotidyltransferase
CD	Connector domain
MT	Methyltransferase
CTD	C-terminal domain
NNI	Non-nucleoside inhibitor
NCBI	National Center for Biotechnology Information
GISAID	Global Initiative on Sharing All Influenza Data
BLAST	Basic local alignment search tool
Cryo-EM	Cryogenic electron microscopy
MOE	Molecular Operating Environment
GFP	Green fluorescent protein
DMSO	Dimethyl sulfoxide
DMEM	Dulbecco’s Modified Eagle Medium
FBS	Fetal bovine serum
